# Turnover rates of photosynthesis determined by fluorescence kinetic analysis

**DOI:** 10.1007/s11120-026-01236-y

**Published:** 2026-09-26

**Authors:** Maxim Y. Gorbunov

**Affiliations:** Environmental Biophysics and Molecular Ecology Program, Department of Marine and Coastal Sciences, Rutgers, the State University of New Jersey, New Brunswick, NJ USA

**Keywords:** Variable fluorescence, Kinetic analysis, Turnover time of photosynthesis, Absolute electron transport rates, Primary production

## Abstract

Maximum turnover rates of photosynthesis impose an upper limit on global primary production, yet traditional estimation methods remain tedious and prone to significant uncertainty. This research presents a new method and operational instrumentation to deduce the maximum turnover rates of photosynthesis from instantaneous kinetic analysis of chlorophyll fluorescence under saturating background light. Measurements of maximum turnover rates in model species of green algae and diatoms over a range of environmental conditions revealed that the rates vary from 150 to 600 s^− 1^ per PSII. These rates correspond to the minimum turnover times of 1.6 to 7 ms. Decreases in turnover rates are driven by low-light photoacclimation and nutrient limitation. In natural populations of near-surface phytoplankton in tropical and subtropical regions, the maximum turnover rates may reach approximately 800 s^− 1^, reflecting photoacclimation to high solar irradiance. In contrast, in cold polar waters, especially under severe nitrogen limitation, the turnover rates decline to 80 to 140 s^− 1^, highlighting a strong temperature and nutrient dependence. Importantly, variations in these turnover rates with nutrient availability show a strong linear correlation with cell growth rates. This discovery provides an efficient, operational tool to quantify the effect of nutrient limitation on phytoplankton growth rates and productivity in the global ocean.

## Introduction

The turnover time of photosynthesis is the time required for the photosynthetic machinery to process the absorbed photon energy and recover from photoexcitation. The reciprocal time defines the turnover rate. The concept of photosynthetic turnover time emerges from measurements of flash-induced oxygen evolution as a function of interval between flashes (e.g., in a dual-flash protocol) or of the frequency of flashes (in a multi-flash protocol) (Heron and Mauzerall, 1971; Myers and Graham 1971; Diner and Mauzerall 1973). As the interval between flashes decreases, the yield of oxygen evolution decreases, and this dependence reflects the kinetics of photosystem recovery from photoexcitation. The concept of turnover times was pioneered by Emerson and Arnold (1932), though they could not resolve turnover times shorter than 40 ms because of limitations of their apparatus.

Measurements of turnover rates of photosynthesis are fundamentally important for the analysis of photosynthetic processes both in the lab and in the field. The maximum turnover rate imposes an upper limit on photosynthetic rates and thus determines the maximum rate (P^max^) achievable under saturating light. The maximum photosynthetic rates are the key variable that determines global primary production on Earth, including the water-column integrated primary production in the ocean (Behrenfeld and Falkowski 1997).

The turnover times are highly heterogeneous as they reflect the complexity of the electron transport chain and the Calvin-Benson-Bassham (CBB) cycle (Diner and Mauzerall 1973). The maximum turnover time of intersystem electron transport is primarily controlled by the redox state of the plastoquinone (PQ) pool, the pool of electron carriers between PSII and PSI (Diner and Mauzerall 1973; Haehnel 1984). The turnover rates are fastest under low irradiance, when the PQ pool remains oxidized, and slow down severalfold under high light, as the PQ pool becomes more reduced (Diner and Mauzerall 1973). On the other hand, the analysis of electron-transport components, PSII, PSI and RUBISCO concentrations within the cell revealed a strong correlation between the ratio of RUBISCO to PSII and turnover rates, suggesting that the carbon fixation cycle is the rate reaction limiting step in light-saturated photosynthesis in algae (Sukenik et al. 1987; Zorz et al. 2015).

Flash-induced O_2_ measurements are tedious and exhibit inherently low sensitivity that preclude them from being used in natural phytoplankton populations. An alternative, more sensitive approach to estimate turnover times relies on using chlorophyll *a* (Chl-*a*) fluorescence. For instance, the maximum turnover rate (1/τ) can be estimated from the analysis of light curves (photosynthesis-versus-irradiance curves) and the functional absorption cross section of PSII (σ_PSII_) (Falkowski and Raven 2007):

1 $$1/\tau = {\rm{ }}{E_k} \times \sigma_{PSII}$$

where E_k_ is the light-saturation parameter of the light curve, i.e., the light intensity at which photosynthetic performance turns from light-limiting to light-saturated regimes. When electron transport rates (ETR) in PSII are deduced in absolute units, the maximum ETR determines 1/τ.

Recording the light curves, especially “slow light curves”, is time-consuming and requires prolonged pre-adaptation to low light in order to accurately reconstruct both the initial slope of the curve and the absolute maximum rates at high light. Classical amplitude-based measurements of photosynthetic rates rely on the discovery that the effective quantum yield of photochemistry in PSII under background light can be deduced from a relative change in fluorescence amplitude (ΔF’/F_m_’) induced by a short saturating flash of light (Genty et al. 1989, 1990). Biophysical models to estimate the absolute ETR in PSII require accurate measurements of PSII absorption, σ_PSII_, (Ley and Mauzerall 1982) and include many variables, which makes their output subject to errors (Schuback et al. 2021; Gorbunov and Falkowski 2022).

A kinetic analysis is the most accurate and direct way to measure the rates of chemical reactions (Semenov 1956 Nobel Prize). This analytical method relies on quantitative time-resolved observation of how the concentrations of the reaction products change over time. This type of analysis can be applied to observe the kinetics of Q_A_ re-oxidation in PSII (i.e., transition Q_A_^−^ → Q_A_) (Mauzerall 1972; Crofts and Wraight 1983; Kolber et al. 1998). Because the redox state of this Q_A_ acceptor imposes the major control on the quantum yield of Chl-*a* fluorescence, the kinetics of Q_A_ re-oxidation is directly derived from the relaxation kinetics of fluorescence yield following a saturating single turnover flash, using Fast Repetition Rate (FRR) (Kolber et al. 1998) or Fluorescence Induction and Relaxation (FIRe) techniques (Gorbunov and Falkowski 2004). The use of fluorescence kinetic analysis implemented in FIRe instruments dramatically improves the accuracy of measuring the absolute ETR in algae, as evident from high correlation with cell growth rates (Gorbunov and Falkowski 2021). Yet, a standardized framework leveraging this kinetic analysis to explicitly isolate the *maximum* turnover rate under operational environmental conditions has not been fully realized.

The objective of this study is to bridge this gap by evaluating fluorescence kinetics as a direct tool to deduce photosynthetic turnover rates. To achieve this objective, we employ a kinetic analysis rooted in the classical physical chemistry frameworks established by Nikolay Semenov (1956). The basic hypothesis of this paper is that the fluorescence relaxation kinetics following a single turnover flash recorded under saturating background light directly reveals the maximum turnover rate of photosynthesis. By validating this hypothesis, this paper introduces a rapid, highly accurate, and field-deployable method and operational instrumentation to determine turnover rates, offering a scalable approach to assess global primary productivity.

## Methods and materials

### Mini-FIRe fluorometers and measurement protocols

Variable fluorescence characteristics are measured with a miniaturized Fluorescence Induction and Relaxation System (called mini-FIRe) (Gorbunov and Falkowski 2021). This instrument is conceptually similar to previous FRR (Kolber et al. 1998) and FIRe systems (Gorbunov and Falkowski 2004), but exhibit ~ 20-fold higher sensitivity and signal-to-noise ratio, compared to its predecessors. High signal-to-noise ratio is crucial for precise analysis of fluorescence relaxation kinetics (Gorbunov and Falkowski 2021). Also, mini-FIRe instruments incorporate a new protocol and mathematical procedure for kinetic analysis of Q_A_ reoxidation rates recorded under background actinic light (as described below).

The peak excitation intensity of the mini-FIRe instrument is optimally adjusted to ensure closure of PSII reaction centers and saturation of fluorescence within approximately 100 µs (i.e. a single electron turnover in PSII). To record variable fluorescence signals under background light, including light curves, the mini-FIRe fluorometer incorporates an actinic light source that is controlled by the FIRe computer. This light source provides uniform illumination of the entire volume of the sample cuvette, which is critical for accurate recording of slow photosynthesis-versus-irradiance curves. These curves can be recorded, in a fully automatic mode, on discrete samples or during underway continuous sampling at sea in a stop-flow regime.

The induction of fluorescence yield during a saturating single-turnover flash and its subsequent relaxation kinetics were analyzed using the mathematical procedure described in (Gorbunov and Falkowski 2021). The FRR model (Kolber et al. 1998; Schuback et al. 2021) was also used for Q_A_ reoxidation kinetics in order to compare the FRR and mini-FIRe relaxation kinetic analysis routines.

### Functional absorption cross-sections of PSII

The functional absorption cross-section of PSII (σ_PSII_) is a product of the optical absorption cross section of PSII (i.e., the physical size of the PSII antennae) and the quantum yield of photochemistry in PSII (Ley and Mauzerall 1982). σ_PSII_ is calculated from the rate of fluorescence induction during the single turnover flash (Kolber et al. 1998; Gorbunov and Falkowski 2004, 2021).

### The photosynthetic turnover time

The photosynthetic turnover time (τ) is defined as the time required for the products of primary photochemical reactions (i.e., electrons produced in PSII as a result of charge separation) to complete the whole cycle of transfer from reaction centers to RUBISCO and the CBB cycle (Herron and Mauzerall 1971; Myers and Graham 1971). The reciprocal turnover time (1/τ) is the turnover rate.

### Analysis of fluorescence relaxation kinetics

The kinetics of electron transport on the acceptor side of PSII (i.e., Q_A_ oxidation) is assessed from analysis of the relaxation kinetics of fluorescence yield following a saturating single turnover flash (Kolber et al. 1998; Gorbunov and Falkowski 2004). This kinetics is highly heterogeneous. It consists of several components because the rate of Q_A_ re-oxidation depends on the state of the second quinone acceptor, Q_B_, which is a mobile two-electron acceptor (Bowes and Croft, 1980; Crofts and Wraight 1983):

2 $${Q_A}^ - {Q_B} \to {Q_A}^{}{Q_B}^ - (200\,{\rm{to}}\,400\,\mu s)$$

3 $${Q_A}^ - {Q_B}^ - \to {Q_A}{Q_B}^ = (600\,{\rm{to}}\,1000\,{\rm{\mu s}})$$

4 $${Q_A}^ - \_ \to {Q_A}^ - {Q_B} \to {Q_A}^{}{Q_B}^ - ( > \sim 2000\,\mu s)$$

Reaction (4) corresponds to the conditions when the Q_B_ is initially out of its binding site on the D1 protein. Also, a fraction of inactive reaction centers with impaired electron transport contributes to the slowest component in the relaxation kinetics. The Q_A_ reoxidation kinetics gets slower when the PQ pool becomes reduced, e.g., under high background light. Also, under ambient light, the speed of Q_A_ reoxidation is indirectly affected by the reactions further down PSII and the PQ pool, including the state of the CBB carbon fixation cycle (see below).

The relaxation kinetics is routinely analyzed using a 3-component analysis, and the average time constant for Q_A_ reoxidation (reported below as *τ*) is calculated as the average time constant for the two fastest components (τ_1_ and τ_2_):

5 $$\tau = {\rm{ }}({\alpha _1}{\tau _1} + {\alpha _2}{\tau _2})/({\alpha _1} + {\alpha _2})$$

Assuming that the slowest component (τ_3_) reflects the processes in inactive centers, the average time *τ* is determined by the rate of Q_A_ re-oxidation in active reaction centers. This average time reflects the actual mixture of the processes described by Eq. 2 to 4. The use of a more complex analysis, e.g., more than 3 components, might potentially allow for better deconvolution of the individual processes, but such analysis requires an extremely high signal-to-noise, which is impractical, especially in field applications.

### Turnover rates from light curves of absolute ETR

The maximum turnover rates are estimated as the maximum absolute ETR rates under saturating background light, deduced from the light curves of variable fluorescence. The “slow light curves” are recorded with sufficient adaptation (~ 30 to 60 s) of the cells to each irradiance level.

Absolute ETR rates per PSII reaction center, as a function of background irradiance, are deduced as described in (Gorbunov and Falkowski 2022):

6 $$ET{R_{Fv}} = E{\sigma _{PSII}}\left[ {(\Delta F'/{F_m}^\prime )/\left( {{F_v}/{F_m}} \right)} \right]$$

where *E* is the photon flux density of background ambient light, *F*_v_/*F*_m_ is the maximum quantum yield of PSII photochemistry in the dark-adapted state, Δ*F*’/*F*_m_’ is the actual quantum yield of PSII photochemistry at a given background light level, and σ_PSII_ is the functional absorption cross section of PSII in dark-adapted state. Δ*F*’/*F*_m_’ is an irradiance-dependent parameter.

### Turnover rates from Q_A_ reoxidation kinetics under background light

Turnover rates of Q_A_ reoxidation are retrieved as described in (Gorbunov and Falkowski 2021). The maximum turnover rate of photosynthesis is determined from the kinetics of Q_A_ reoxidation following a saturating single turnover flash measured under steady-state saturating background light.

### Measurements of the redox state of the PQ pool

Alterations in the redox state of the PQ pool with background irradiance are assessed as described in (Levitan et al. 2015) The redox state is deduced from the analysis of fluorescence induction curves on the millisecond time scale in response to a saturating multiple turnover flash of 200 ms duration. The fraction of the oxidized PQ molecules (noted as PQ_ox_) is estimated from a decrease in the area above the normalized induction curve relative to the reference area measured for fully oxidized PQ pool (Levitan et al. 2015). This reference area is calculated from the induction curve recorded after a short (1s) period of darkness followed by a short (1 s) pre-exposure to far-red actinic light (the wavelengths > 700 nm) to fully oxidize the PQ pool.

### Strains and culture conditions

Algal cultures were grown at 18 °C in F/2 medium under continuous white light (25 to 600 µmol photons m^− 2^ s^− 1^) from light-emitting diode lamps. The cultures included a diatom (*Thalassiosira pseudonana CCMP1335*), and a chlorophyte (*Dunaliella tertiolecta CCMP1320*).

### Nutrient stress experiments

Nutrient stress experiments are conducted on batch cultures growing in low-nitrogen media under high light (500 µmole quanta m^− 2^ s^− 1^) as described in (Gorbunov and Falkowski 2021). First, cultures were pre-acclimated (for 4–5 days) to low-nitrogen F/2 media, with [NO_3_] of 10 µM, which is 90 times lower than in normal F/2 media. This pre-acclimation allowed the cells to maintain maximum growth rates even in this low-nitrogen media, although the cellular Chl-*a* content reduced severalfold. Then the slow progression of the nutrient stress was monitored in batch cultures as the cells uptook the remaining nitrate and the nitrogen concentration in the media became undetectable within 48–72 h.

### Growth rates measurements

Instantaneous growth rates (µ) are determined from an increase in cell density (N, cell/mL) over a short (4 to 6 h) period of time (Δt), using an exponential growth equation:

7 $${\rm{\mu }}\,{\rm{ = }}\,{\rm{ln[N}}\,{\rm{(t}}\,{\rm{ + }}\,{\rm{\Delta t)/N}}\,\left( {\rm{t}} \right){\rm{]/\Delta t}}$$

Cell densities were recorded using Beckman Coulter Multisizer 3 cell counter.

## Kinetic analysis of fluorescence relaxation and Q_A_ reoxidation rates

**Fig. 1:**
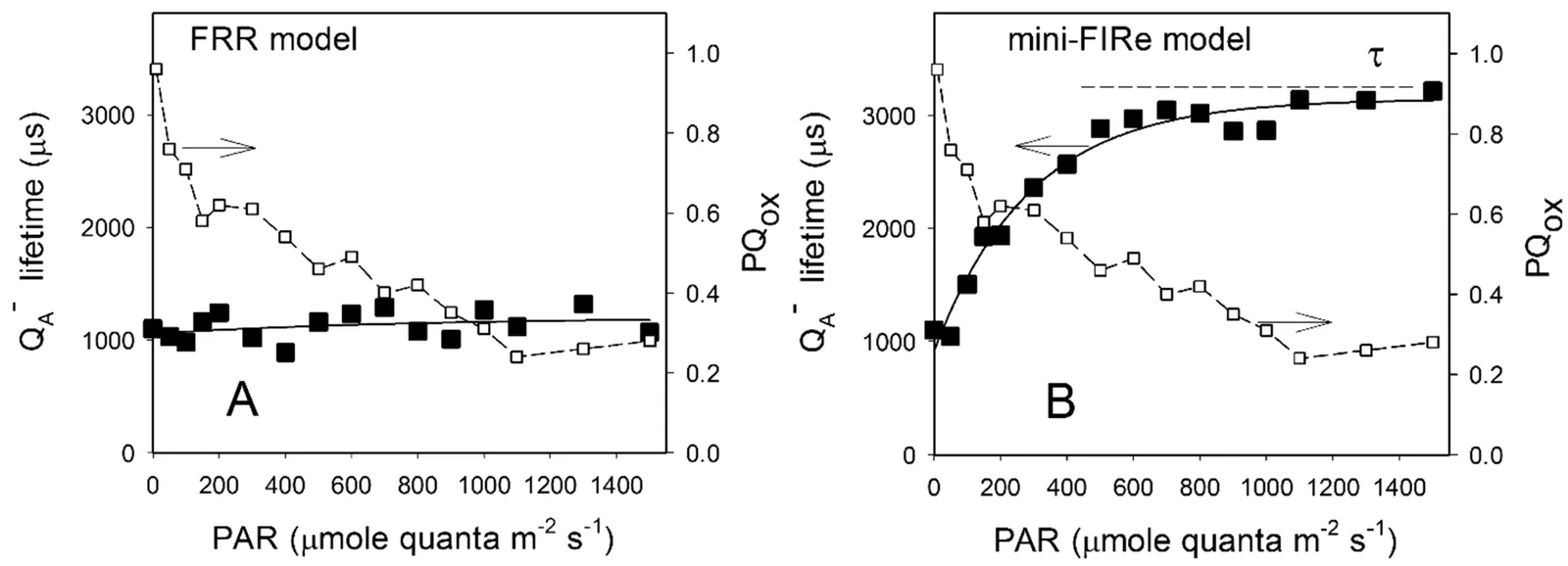
Influence of steady-state background PAR on Q_A_ reoxidation turnover times (closed dots), relative to the redox state of the PQ pool (PQ_ox_, open dots). Turnover times were retrieved from the analyses of fluorescence relaxation kinetics following a saturating single-turnover flash in the green algae, *Dunaliella tertiolecta*. Two mathematical models were used for these kinetic analyses: the FRR model (**A**) and the new FIRe model (**B**). Note that the FRR model fails to capture actual changes in Q_A_ reoxidation rates as the PQ pool becomes progressively reduced under higher PAR levels

The FRR technique (Kolber et al. 1998) has been developed to quantify fluorescence induction induced by a saturating single turnover flash and subsequent relaxation of fluorescence which reflects the rates of Q_A_ reoxidation. It was expected that as the background PAR increases and the PQ pool becomes reduced, the actual speed of electron flow would slow down and the speed of FRR relaxation kinetics would decrease. Unexpectedly, the FRR relaxation analysis showed little change or even decline in the measured fluorescence relaxation times with an increase in PAR (Fig. 1A). Such pattern was observed in all algal cultures and natural samples of phytoplankton (Gorbunov and Falkowski 2021). These observations demonstrate that the FRR relaxation analysis does not reflect real photosynthetic rates achieved under ambient (or background) light.

Why does the FRR relaxation kinetic model fail to capture the actual photosynthetic rates under background light? The FRR model was originally developed for analysis of variable fluorescence in dark-adapted samples (Kolber et al. 1998). This model and mathematical formalism (Kolber et al. 1998; Schuback et al. 2021) do not include the effects of background light on the recorded fluorescence relaxation kinetics. As background light pumps energy into photosystems and stimulates the dynamic closure of a fraction of reaction centers, this process competes with the Q_A_ reoxidation and inevitably distorts the observed fluorescence relaxation kinetics (Gorbunov and Falkowski 2021).

To overcome this limitation of the FRR biophysical model, a new mathematical model and data analysis procedure have been developed to take into account the effects of background light on fluorescence relaxation kinetics, and this procedure has been implemented in mini-FIRe instruments (Gorbunov and Falkowski 2021).

**Fig. 2:**
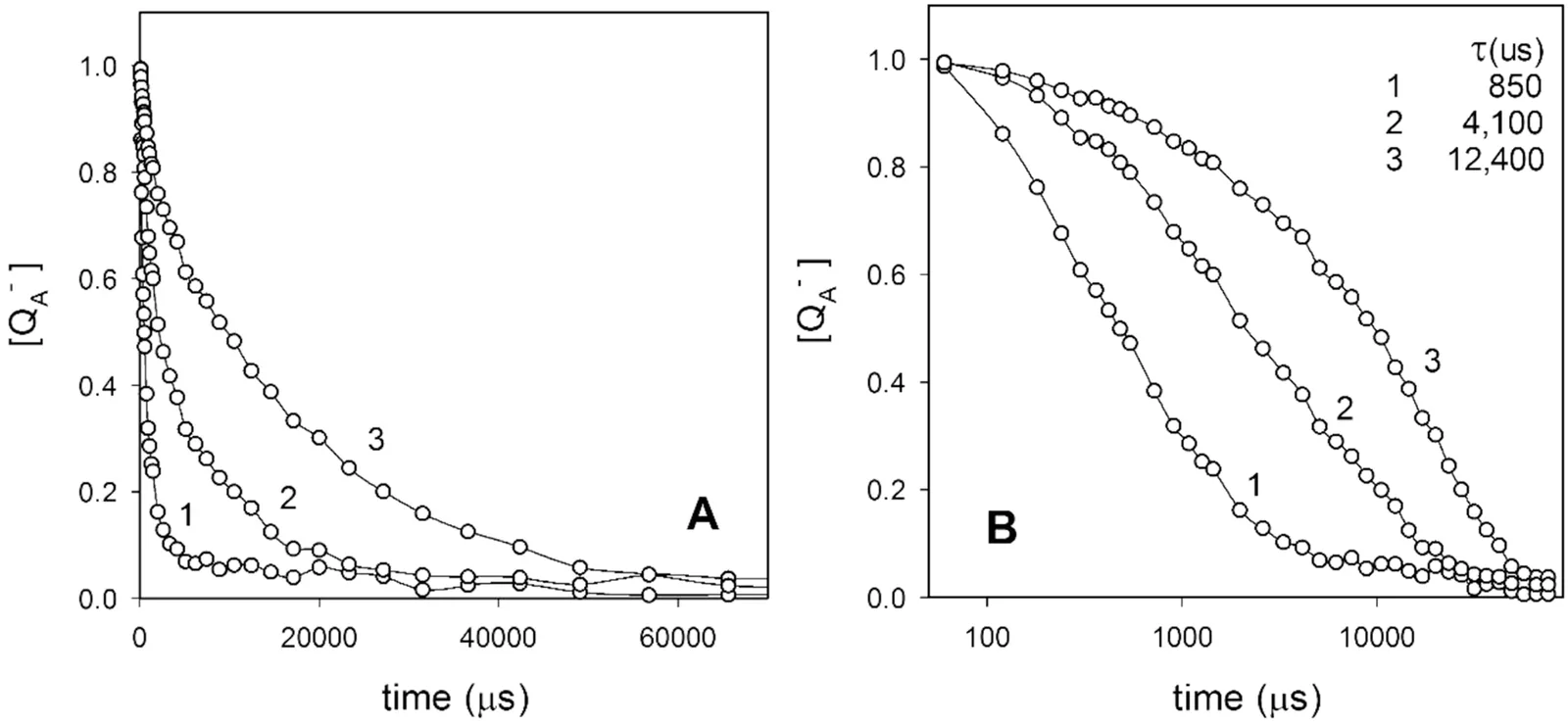
Kinetics of Q_A_ re-oxidation in *Dunaliella t*. The kinetic profiles were derived from chlorophyll fluorescence relaxation kinetics, following a single-turnover flash, and displayed on linear (**A**) and logarithmic (**B**) time scales. The profiles were taken under different metabolic states: (1) dark measurement on low-light acclimated alga, (2) under steady-state saturating background light with a fully active CBB cycle, (3) under saturating light in cells with a suppressed CBB cycle

The new kinetic analysis, using a mini-FIRe instrument, revealed a strong effect of background PAR on the retrieved rates of Q_A_ reoxidation (Figs. 1B and 2). At PAR = 0, the PQ pool was oxidized (PQ_ox_ = 1) and the time of Q_A_ reoxidation was minimal (Fig. 1B and profile (1) in Fig. 2). As PAR increased and the PQ pool became more reduced, the time of Q_A_ reoxidation increased, reaching a plateau at saturating PAR levels. The reciprocal of this time measured at steady-state saturating PAR determined the maximum turnover rate of photosynthesis (Gorbunov and Falkowski 2021). These data indicate that the maximum turnover rate of photosynthesis can be determined from an instantaneous measurement of fluorescence relaxation kinetics under steady-state saturating PAR, without recording the whole P-E light curve.

Under saturating background light, the rate of Q_A_ reoxidation is controlled by the redox state of the PQ pool and indirectly modulated by the activity of the CBB carbon fixation cycle (Fig. 2). When the CBB cycle was suppressed via 30 min of dark adaptation, the measured turnover time increased approximately 3-fold compared to the fully activated CBB cycle (profile 3 versus profile 2 in Fig. 2). The kinetic profile with the suppressed CBB cycle was recorded immediately (within ~ 1 s) following the dark-to-high light transition. Similarly, chemical inhibition of the CBB cycle using 10 mM glycoladehyde induced a comparable increase in the turnover time. Note that the Q_A_ reoxidation kinetics measured in dark (profile 1 in Fig. 2) remained unaffected by the state of the CBB cycle.

**Fig. 3:**
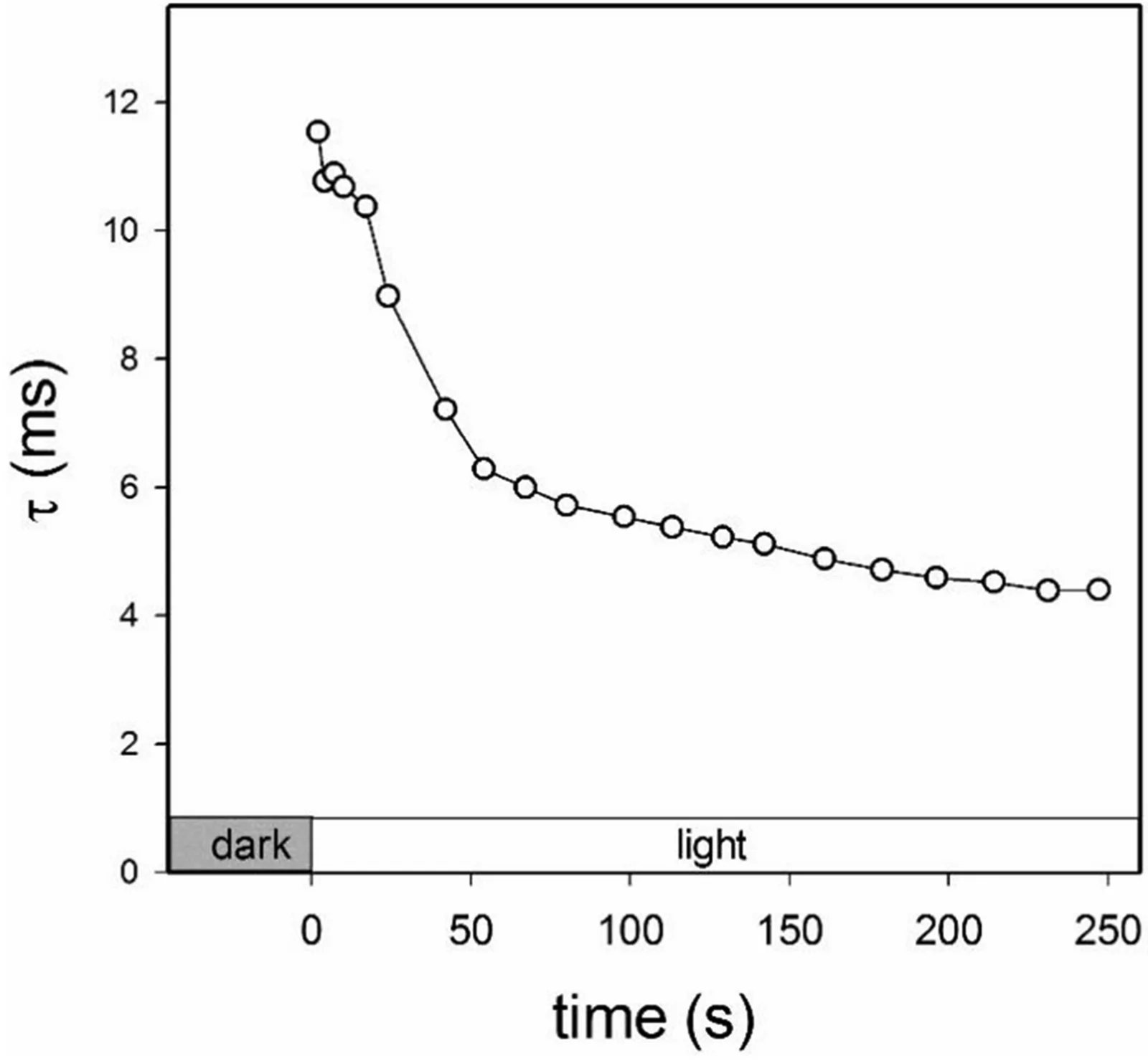
Dynamics of photosynthetic turnover time (τ) during the dark-light transition in *Dunaliella t.*. Cells were dark-adapted for 30 min and exposed to saturating light (1000 µmol m^− 2^ s^− 1^). Values of τ were derived from Q_A_ reoxidation kinetics induced by a single-turnover flash under saturating background light. The dynamics τ reflects the light-dependent activation and recovery of the CBB cycle from a dark-suppressed to fully active state

Further evidence about the influence the CBB cycle metabolic state on the photosynthetic turnover time comes from the dynamics of τ during the dark-light transition (Fig. 3). Because prolong dark adaptation dramatically increases these turnover times and requires several minutes of light exposure for full recovery (Fig. 3), pre-measurement dark adaptation must be avoided when assessing steady-state fluorescence and photosynthetic light curves. Instead, low-light adaptation is recommended; this approach accelerates the relaxation of non-photochemical quenching (NPQ) and photoinhibition, particularly in cells recently exposed to high-light environments (e.g., daytime phytoplankton samples collected from the upper ocean).

### Influence of growth irradiance on turnover rates of photosynthesis

**Fig. 4:**
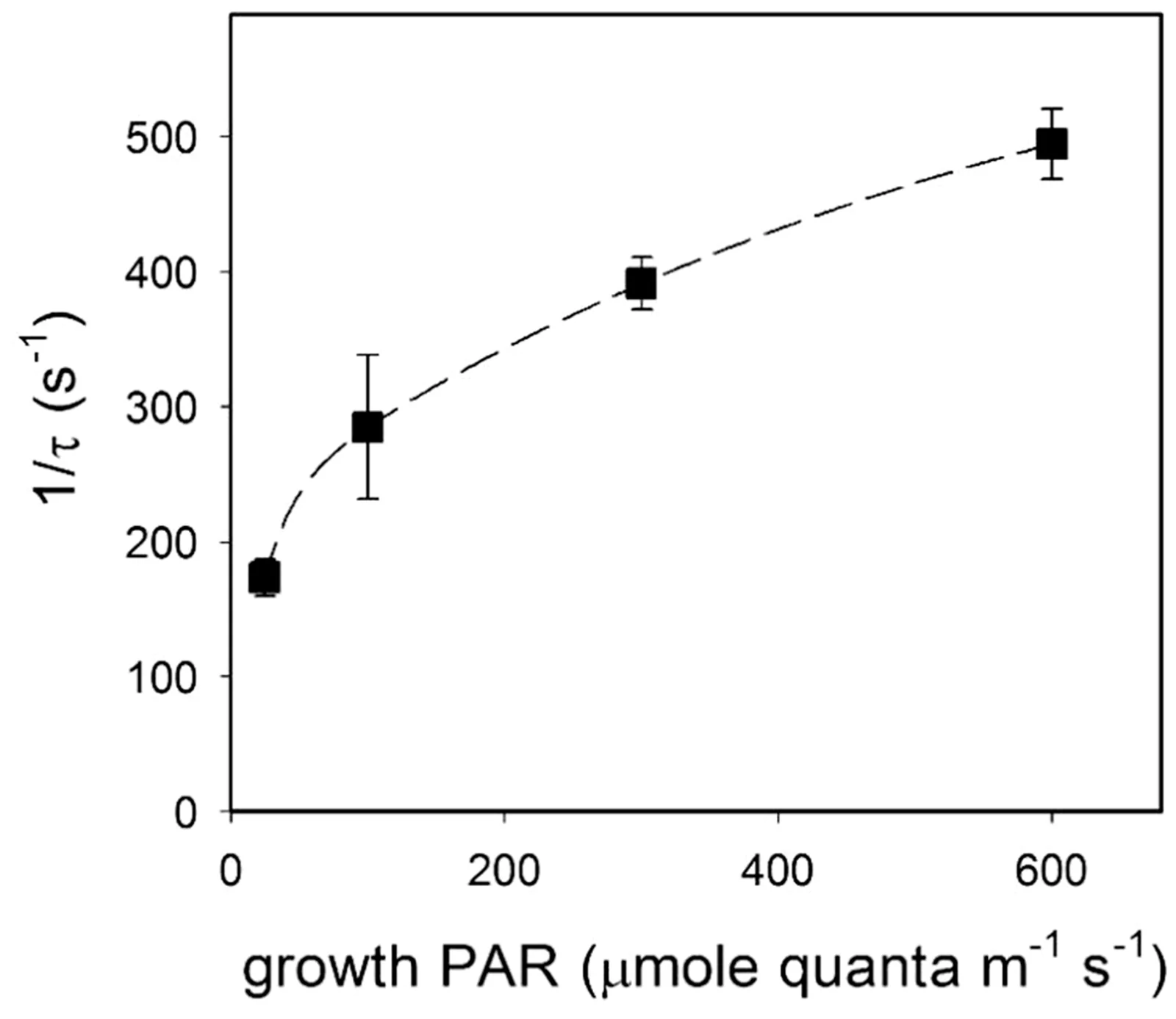
Effect of growth irradiance on maximum turnover rates of photosynthesis in *Dunaliella t.* The cultures were grown under nutrient replete conditions and continuous light

The turnover rates of photosynthesis decrease markedly at low growth irradiance (Fig. 4), reflecting a classical pattern of photoacclimation.

**Fig. 5:**
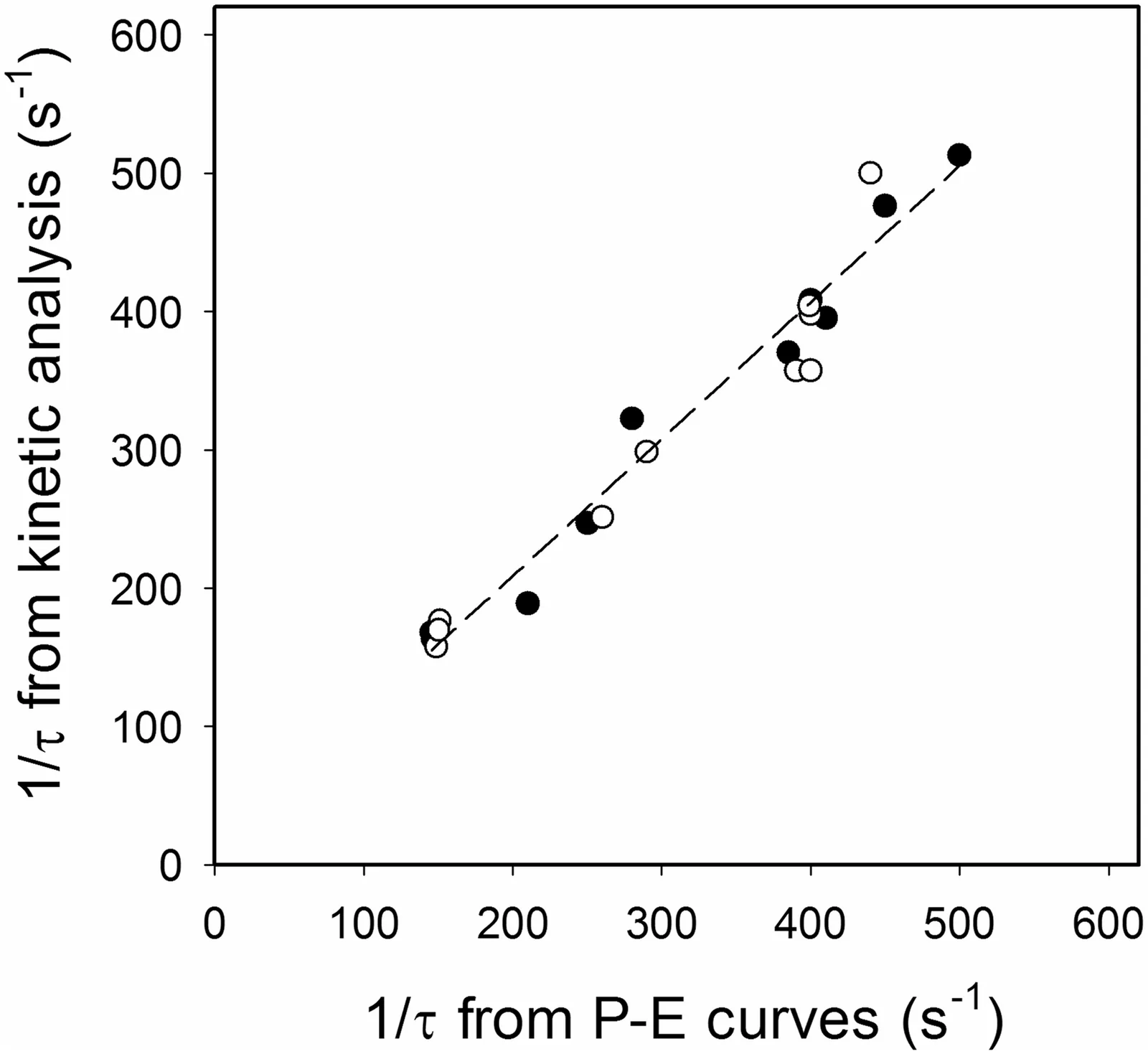
The relationship between two independent estimates of the maximum turnover rates of photosynthesis. The rates were derived from the light curves of variable fluorescence and from the kinetic analysis of Q_A_ reoxidation under saturating light. The measurements were conducted in nutrient-replete cultures of the green algae, *Dunaliella tertiolecta*, (closed dots) and the diatom, *Thalasiosira pseudonana* (open dots), grown under different irradiance levels. The increase in turnover rates from 150 to 500 s^− 1^ per PSII is driven by photoacclimation from low to high growth light

The growth irradiance dependence is evident from two alternative methods for estimating the turnover rates. Figure 5 shows a high correlation between the maximum turnover rates determined from the classical light curves and from the kinetic analysis of Q_A_ reoxidation under saturating PAR. It should be noted that these two independent methods produced virtually identical values when cells were grown under nutrient replete conditions (Fig. 5). Nutrient limiting conditions deteriorate this correlation markedly, because the classical amplitude-based method for ETR becomes less accurate (Gorbunov and Falkowski 2021).

### Diel cycles of turnover rates

When algae are grown under natural day/night cycles of irradiance (both in the lab or in natural phytoplankton populations), they exhibit strong diel cycles of turnover rates, with the rates being maximal during the day and minimal (ca. 30–50% decrease) at night (e.g., Sherman et al. 2020). This diel pattern is consistent with the diel patterns of carbon fixation rates in phytoplankton (Harding et al. 1981; Prezelin 1992). The exposure of cells taken at night to daytime light does not restore (at least within ~ 30 min of light exposure) the high daytime values, suggesting that this diel cycle is driven by the circadian rhythm of cellular metabolism.

### Effect of nutrient limitation on turnover rates

Photosynthesis in the ocean is fundamentally limited by the availability of nutrients, such as nitrogen and iron, and in some regions co-limited by phosphorus (Falkowski and Raven 2007). This global nutrient paucity severely impairs the efficiency of oceanic photosynthesis, causing phytoplankton communities to operate at approximately 50% of their maximum capacity (Lin et al. 2016).

**Fig. 6:**
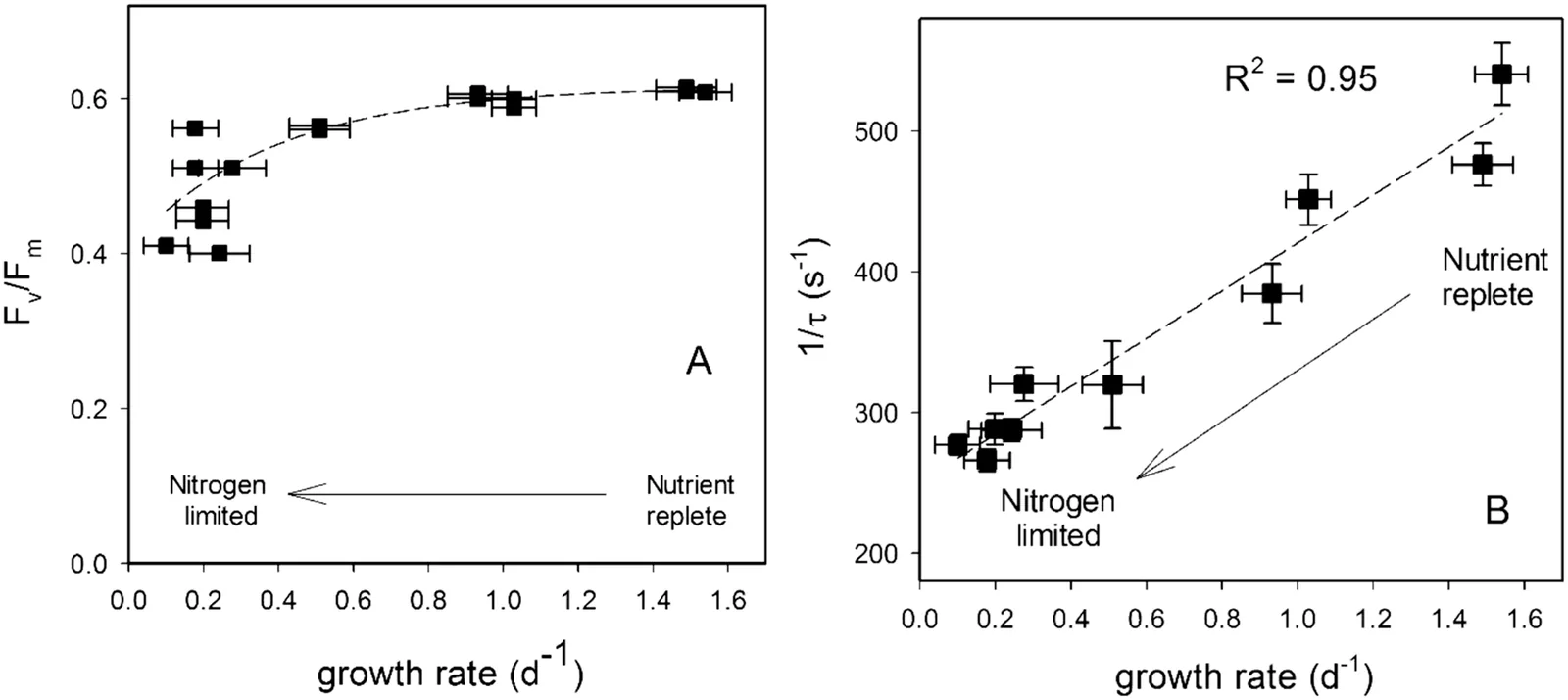
Effect of nitrogen limitation on (**A**) the quantum yield of photochemistry in PSII and (**B**) the maximum turnover rates of photosynthesis in *Dunaliella t.*, relative to cell growth rates

Variable fluorescence and more specifically the F_v_/F_m_ ratio is commonly used as a diagnostic of unfavorable environmental factors, including nutrient limitation, on photosynthesis (Papageorgiou and Govindjee 2004). F_v_/F_m_ is usually reduced under nutrient stress, such as nitrogen or iron limitation (Gorbunov and Falkowski 2022). However, in case of nitrogen limitation, the relationship between F_v_/F_m_ and the severity of nutrient stress is highly nonlinear and depends on how the cells are exposed to nutrient stress (Kolber et al. 1988; Parkhill et al. 2001 and Fig. 6A). Consequently, F_v_/F_m_ can remain deceptively elevated even when cell division and growth rates are severely suppressed (Fig. 6A), highlighting its limitations as a standalone quantitative proxy.

To address this diagnostic limitation, alternative biophysical indicators have been evaluated. Comparative analysis of the green alga *Dunaliella t.* under controlled nitrogen limitation demonstrates a distinct divergence between F_v_/F_m_ (Fig. 6A) and the maximum photosynthetic turnover rate (1/τ. Figure 6B) relative to growth rates. Unlike the non-linear response of F_v_/F_m_, the reduction in 1/τ exhibits a robust, linear correlation with declining growth rates (Fig. 6B). These empirical findings demonstrate that the maximum turnover rate (1/τ) provides a significantly more sensitive and reliable quantitative diagnostic for monitoring and quantifying nitrogen stress in marine phytoplankton.

## Discussion and concluding remarks

The data presented here revealed that the maximum turnover rate of photosynthesis (1/τ) can be determined from an instantaneous measurement of fluorescence relaxation kinetics induced by a single turnover flash when such a measurement is conducted under steady-state saturating background light. This determination of 1/τ can be done without recording the whole P-E light curve. This result corroborates the previous development of the kinetic-based analysis of absolute ETR rates (Gorbunov and Falkowski 2021) that dramatically improved fluorescence-based measurements of photosynthetic rates.

While the Q_A_ reoxidation rate directly reflects intra-PSII electron flow velocity, it is tightly coupled to downstream metabolic processes. Under saturating background light, suppressing CBB cycle activity induced a sharp decline in Q_A_ reoxidation kinetics (Fig. 2, profile 3, compared to 2). This strong feedback inhibition confirms that these measured kinetic rates reliably index the absolute dynamic turnover of overall photosynthesis.

Beyond primary CO_2_ fixation within the CBB cycle, alternative electron sinks, including the Mehler reaction and associated NADPH-consuming pathways, can also influence overall photosynthetic turnover rates. Because the engagement of these alternative pathways depends on taxonomic affiliation and environmental conditions, their precise impact remains an important subject for future research.

Measurements of maximum turnover rates in model algal species over a range of environmental conditions revealed a wide range of these rates from 150 to 600 s^− 1^ per PSII (Figs. 4 and 5). These rates correspond to the minimum turnover times of 1.6 to 7 ms, which is consistent with previous estimates using the O2 evolution technique (Sukenik et al. 1987; Zorz et al. 2015).

In natural populations of near-surface phytoplankton in tropical and subtropical regions, the maximum turnover rates reach even higher values of approximately 800 to 900 s^− 1^ per PSII, reflecting photoacclimation to high solar irradiance (Sherman et al. 2022). In contrast, in cold polar waters, especially under severe nitrogen limitation, the turnover rates decrease down to 80 to 140 s^− 1^ per PSII (Ko et al. 2020). This decrease is consistent with the temperature dependence of primary production rates in phytoplankton (Behrenfeld and Falkowski 1997; Kulk et al. 2012) and the effect of nutrient stress (Gorbunov and Falkowski 2021).

### Importance of fluorescence measurements under saturating background light to deduce the maximum turnover rates of photosynthesis

The new operational protocol implemented in mini-FIRe instruments to deduce the kinetics of Q_A_ reoxidation under background light revealed that the speed of electron transfer and thus the actual turnover rates decrease markedly with ambient PAR levels (Fig. 1B). This process reflects the gradual reduction of the PQ pool, which is consistent with the modern concept that the redox state of the PQ pool redox is the primary control of electron flow between PSII and PSI (Diner and Mauzerall 1973; Haehnel 1984).

Depending on the environmental conditions (such as photoacclimation status and nutrient availability), the difference between the Q_A_ reoxidation rate in dark and that under saturating background light may vary severalfold, with no correlation between these two rates. Fluorescence kinetics recorded in a dark-adapted state is fast (with typical times of ~ 500 µs) and does not reflect the maximum turnover rate achievable under saturating light. For instance, the rate of Q_A_ reoxidation recorded in dark (profile 1 in Fig. 2) is not affected by the state of the CBB cycle. Also, the dark rates of Q_A_ reoxidation are not affected by nitrogen limitation or photoacclimation (Falkowski et al. 1986; Gorbunov and Falkowski 2022), but the maximum turnover rates achieved at saturating light vary dramatically (Gorbunov and Falkowski 2021 and this research). Therefore, it is crucially important to measure the fluorescence relaxation kinetics under steady-state saturating background light in order to derive the maximum turnover rates of photosynthesis.

### Using fluorescence kinetic analysis for marine and terrestrial plants

Over the past three decades, variable fluorescence has become a cornerstone methodology in plant physiology and oceanography (Papageorgiou and Govindjee 2004). While the high pigment density characteristic of terrestrial leaves enhances fluorescence signals and optimizes the signal-to-noise ratio, it simultaneously introduces confounding optical artifacts. Specifically, the pigment packaging effect induces self-shading and fluorescence reabsorption, rendering accurate quantifications of the functional absorption cross-section of PSII (σ_PSII_) and absolute electron transport rates virtually impossible in intact leaves. Consequently, ETRs are conventionally reported in relative units (Schreiber et al. 1986; Schreiber 2004), a limitation that impedes direct quantitative comparisons across samples with varying pigment densities. Kinetic analysis of photosynthetic turnover rates resolves this fundamental constraint of amplitude-based fluorescence techniques, as kinetic profiles remain unaffected by packaging-induced optical artifacts.

By leveraging instantaneous measurements under ambient light conditions, kinetic analysis circumvents the need for dark adaptation and time-consuming light-curve recordings. This capacity for rapid, absolute maximum ETR quantification provides a critical methodological advantage for in situ aquatic field sampling of macrophytes and corals utilizing specialized instrumentation, such as the diving FIRe system (Gorbunov and Falkowski 2021).

### Overcoming limitations of ^14^C uptake measurements

In marine ecosystems, primary production rates have traditionally been measured by following the uptake of radioactive inorganic carbon ^14^C into phytoplankton cells. This method, pioneered by (Steemann Nielsen 1952), is extremely sensitive, but requires incubating the samples in a confined space. Incubation of samples in bottles, especially in the oligotrophic open ocean, could lead to large artifacts due to trace metal contamination (Carpenter and Lively 1980; Fitzwater et al. 1982), and due to alterations in the community structure during longer-term incubations (Eppley 1980). Also, the measured rates of ^14^C uptake depend dramatically on the incubation duration (Marra 2009; Halsey et al. 2011, 2013). The results of short-term ^14^C incubations strongly depend on the extent of nitrogen limitation (Halsey et al. 2011, 2013) - they reflect gross production rates in nutrient replete phytoplankton and net production rates in nutrient limited plankton. The difference between gross and net production rates may reach as much as > 100%, thus introducing large uncertainties to the interpretation of ^14^C incubations, if the extent of nutrient stress is not a priori known (Halsey et al. 2011; Milligan et al. 2015). The fluorescence kinetic analysis offers an easily measured quantitative index of nutrient stress (Gorbunov and Falkowski 2021) that is critically needed for accurate interpretation of ^14^C uptake measurements when the severity of nutrient stress varies.

### Estimating the electron requirements of carbon fixation from kinetic fluorescence analysis

Converting fluorescence-based electron transport rates (ETR) into carbon fixation rates requires an accurate quantification of the electron requirement for carbon fixation — defined as the number of electrons generated by photosystem II (PSII) to fix a single molecule of CO_2_. In aquatic ecosystems, this requirement can vary severalfold due to fluctuating environmental conditions, most notably the severity of nutrient limitation (Lawrenz et al. 2013; Hughes et al. 2018; Zhu et al. 2017; Ko et al. 2020). However, assessing maximum photosynthetic turnover rates provides a robust, quantitative proxy for estimating these changing electron requirements (Gorbunov and Falkowski 2021). Integrating this proxy significantly enhances the precision of fluorescence-derived estimates of primary production in aquatic environments.

## Data Availability

The data are available from the author upon request.

## Abbreviations

PSII: Photosystem II
ETR: Electron transport rates
Q_A_, Q_B_: Primary and secondary quinone acceptors of PSII
PQ: Plastoquinone
RUBISCO: Ribulose-1,5-bisphosphate carboxylase/oxygenase
CBB: Calvin-Benson-Bassham cycle
1/τ: Maximum turnover rate of photosynthesis
σ_PSII_: The functional absorption cross section of PSII
F_v_/F_m_: Maximum quantum yield of photochemistry in PSII
ΔF'/Fm': Effective quantum yield of photochemistry under background light
FRR: Fast repetition rate fluorometry
FIRe: Fluorescence induction and relaxation technique
PAR: Photosynthetically active radiation of background light
